# The Glycosylation Pattern of Common Allergens: The Recognition and Uptake of Der p 1 by Epithelial and Dendritic Cells Is Carbohydrate Dependent

**DOI:** 10.1371/journal.pone.0033929

**Published:** 2012-03-30

**Authors:** Abeer Al-Ghouleh, Ramneek Johal, Inas K. Sharquie, Mohammed Emara, Helen Harrington, Farouk Shakib, Amir M. Ghaemmaghami

**Affiliations:** School of Molecular Medical Sciences, Division of Immunology, University of Nottingham, Queen's Medical Centre, Nottingham, United Kingdom; Université Paris Descartes, France

## Abstract

Allergens are initiators of both innate and adaptive immune responses. They are recognised at the site of entry by epithelial and dendritic cells (DCs), both of which activate innate inflammatory circuits that can collectively induce Th2 immune responses. In an attempt to have a better understanding of the role of carbohydrates in the recognition and uptake of allergens by the innate immune system, we defined common glycosylation patterns in major allergens. This was done using labelled lectins and showed that allergens like Der p 1 (*Dermatophagoides pteronyssinus* group 1), Fel d 1 (*Felis domisticus*), Ara h 1 (*Arachis hypogaea*), Der p 2 (*Dermatophagoides pteronyssinus* group 2), Bla g 2 (*Blattella germanica*) and Can f 1 (*Canis familiaris*) are glycosylated and that the main dominant sugars on these allergens are 1–2, 1–3 and 1–6 mannose. These observations are in line with recent reports implicating the mannose receptor (MR) in allergen recognition and uptake by DCs and suggesting a major link between glycosylation and allergen recognition. We then looked at TSLP (Thymic Stromal Lymphopoietin) cytokine secretion by lung epithelia upon encountering natural Der p 1 allergen. TSLP is suggested to drive DC maturation in support of allergic hypersensitivity reactions. Our data showed an increase in TSLP secretion by lung epithelia upon stimulation with natural Der p 1 which was carbohydrate dependent. The deglycosylated preparation of Der p 1 exhibited minimal uptake by DCs compared to the natural and hyperglycosylated recombinant counterparts, with the latter being taken up more readily than the other preparations. Collectively, our data indicate that carbohydrate moieties on allergens play a vital role in their recognition by innate immune cells, implicating them in downstream deleterious Th2 cell activation and IgE production.

## Introduction

Allergens are foreign proteins that induce type I hypersensitivity reactions through eliciting Th2 immune responses, which culminate in IgE production and allergy. Epithelial cells are the first line of defence against foreign antigens; they recognise antigens through PRRs like TLRs [1], [2] and through PAR 1-PAR 4 [3], [4]. Ligation of these receptors with microbial motifs or allergens activates innate immune responses, inflammatory signalling pathways and the production of cytokines that direct the Th1/Th2 immune polarization [3], [4]. One key cytokine secreted by epithelial cells in response to allergen exposure is TSLP, an IL-7 like cytokine with a plethora of biological activities [5]. It has recently been shown that high levels of TSLP induce Th2 immune responses in humans by modulating the phenotype of DCs (e.g. up-regulating the expression of OX40L) and by directly acting on activated T cells [6]–[8]. Furthermore, upon TSLP stimulation, DCs were found to produce the Th2 attracting chemokines CCL17 and CCL22 [9], [10] that have been shown to recruit Th2 cells into the airway [10]–[12].

Whilst antigen-epithelial cell interaction leads to conditioning of DCs with knock-on effects on downstream events such as T cell differentiation, DCs act as sentinels of the immune system and are able to recognise antigens at the site of entry through different PRRs such as TLRs, NOD like receptors and C-type lectin receptors [3]. They then present antigens to naive T helper cells in draining lymph nodes leading to T cell differentiation into functionally distinct subsets such as Th1, Th2, Treg and Th17 [3], [12], [13]. Amongst the well-studied PRR expressed by DCs are C-type lectins, such as MR, DC-SIGN, dectin-1, langerin and DEC-205 that are involved in the recognition and capture of many glycosylated antigens as well as self-antigens and pathogens [14]. For example, MR recognises a wide range of both endogenous and exogenous ligands through their carbohydrate moieties [15]–[17], like mannose, fucose and N-acetylglucosamine [18], [19].

It has been suggested that what might differentiate allergens from other non-allergenic proteins lies in their protease activity, surface features and/or glycosylation patterns. These three features either singly or collectively might render some proteins allergenic [20]–[22]. The contributions of protease activity and surface features of allergens to allergenicity have been thoroughly investigated in recent years [20], [23], [24]. The glycosylation pattern of allergens, however, has only recently been considered as a possible distinguishing feature of these proteins. Much of the research in this area has so far only focussed on the quantitative determination of the carbohydrate content of allergens, without much consideration for the whole carbohydrate structure and the pattern of glycosylation or its biological relevance [25]–[27]. It is known that carbohydrate determinants are the most frequently encountered epitope structures for IgE [26], [28], and as such have been named Cross-reactive Carbohydrate Determinants (CCD). These determinants are asparagine linked carbohydrate moieties and they mainly consist of xylose and core-3-linked fucose, which form the vital part of two independent IgE epitopes [28], [29]. These CCDs are mainly found in plants, insects and parasites, but are absent in mammals and are therefore immunogenic [26], [27].

In an attempt to have a better understanding of the role of carbohydrates in allergen recognition by the innate immune system, we examined a number of commonly encountered allergens for their quantitative and qualitative carbohydrate content by using labelled lectins known to react with specific sugar moieties [15]. Having mapped the carbohydrate content of these allergens, we then proceeded to define the influence of these sugar residues on allergen recognition by epithelial cells and DCs. The results obtained underline the pivotal role of carbohydrates in allergen recognition and handling by innate immune cells, and this should now pave the way for instigating novel approaches for controlling allergic sensitizations at the point of initial contact with the immune system.

## Materials and Methods

### Allergen and non allergen protein preparations

Purified natural and recombinant Der p 1, Der p 2, Fel d 1, deglycosylated Fel d 1 (DFel d 1 lacking a major glycosylation site), Can f 1, Ara h 1 and Bla g 2 were purchased from Indoor Biotechnology, Warminster, UK. Papain, Bromelain and Calpain ll were purchased from Sigma. The Staphopain B (StpB) was purchased from Biocentrum Ltd, UK. Cysteine protease B (CPB) was kindly provided by Prof Jeremy Mottram, University of Glasgow (UK).

### Glycosylation analysis

All protein preparations (5 µg per sample) were run on a 12% Novex Tris-Glycine precast gel (Invitrogen, Paisley, UK) prior to being transferred to nitrocellulose membrane following standard procedure. This was followed by detection of different glycans using DIG Glycan Differentiation Kit (Roche, Welwyn Garden City, UK) following the manufacturer's instructions (Table 1).

**Table 1:** Western blot results of different lectin reactions with different allergens.

Allergen	GNA (anti mannose)	DSA	MMA	PNA	SNA	Anti-1,3 Fucose
**nFel d 1**	**+**	**+**	**+++**	**−**	**−**	**−**
**DFel d 1**	**−**	**−**	**+**	**++**	**−**	**−**
**Der p 2**	**−**	**+**	**++**	**+++**	**++**	**+**
**Ara h 1**	**+++**	**−**	**+**	**++**	**++**	**++**
**Bla g 2**	**+++**	**++**	**+**	**++**	**++**	**+++**
**Can f 1**	**+**	**−**	**+**	**−**	**++**	**−**
**rDer p 1**	**+++**	**−**	**−**	**+**	**−**	**−**
**Der p 1**	**+**	**++**	**−**	**++**	**++**	**+++**
**Papain**	**++**	**+**	**−**	**+**	**+**	**++**
**Bromelain**	**+**	**+**	**−**	**++**	**+**	**+++**

+++: strong reaction, ++: moderate reaction, +: mild reaction, −: no reaction. Sugar binding specificities of lectin used for glycosylation analysis are as follows: GNA recognises terminal mannose (1–3), (1–2) and (1–6) linked to Mannose; DSA, recognises galactose (1–4) linked to N-acetylgalactosamine; MAA recognises sialic acid linked (2–3) to galactose; PNA recognises core galactose (1–3) N acetylgalactosamine; SNA, recognises sialic acid linked (2–6) to galactose; Anti 1, 3 Fucose recognises core (1,3) fucose. DSA, Datura stramonium agglutinin; GNA, Galanthus nivalis agglutinin; MAA, Maackia amurensis agglutinin; PNA, peanut agglutinin; SNA, Sambucus nigra agglutinin.

The detection of 1,3 fucose was done using anti-1,3 fucose rabbit polyclonal antibody (no. AS07 268, Agrisera, UK) which cross-reacts with fucose residues bound to N-Glycans in alpha 1,3 in plants and insects. The concentration of antibody used was 1 µg/10 ml of TBS buffer.

### Periodate deglycosylation

Der p 1 preparations were treated with sodium metaperiodate (Sigma) at a molar ratio of 5∶1. The reaction mixture was incubated for 30 and 60 mins at room temperature in the dark. The oxidation process was stopped by adding 0.25 ml ethylene glycol per ml of sample. Samples were then dialyzed at room temperature overnight against PBS. The allergen preparations were then labelled with Lightning-Link FITC Antibody Labelling Kit from Novus Biologicals (Cambridge, UK).

### Coomassie staining analysis

Gels were washed with deionised H_2_O and stained with Coomassie brilliant blue Imperial ready to go stain (Invitrogen) for 1 hr. They were then destained overnight with H_2_O according to the manufacturer's protocol.

### Culturing BEAS-2B epithelial cells with different Der p 1 glycoforms

Human bronchial epithelial cell line BEAS-2B (kindly provided by Professor Ian Hall, University of Nottingham, UK) was used for measuring TSLP production in response to different allergen preparations. Cells were cultured in Dulbecco's Modified Eagle's Medium (Invitrogen), along with 10% low endotoxin foetal bovine serum (Autogen Bioclear, UK), 2 mM L-Glutamine (Sigma) and 1% Penicillin/Streptomycin (Sigma). After reaching confluency, the cells were then trypsinised using 0.25% trypsin-EDTA (Sigma) and incubated for 5 minutes at 37°C, 5% CO_2_. BEAS-2B cells (1×10^6^ cells/ml) were added to 24-well plates (Corning), together with either 1 µg/ml of natural Der p 1 (Indoor Biotechnology) or periodate deglycosylated natural Der p 1 (1 µg/ml). All cultures received 50 ng/ml LPS (sigma). Plates were then incubated at 37°C, 5% CO_2_ for 24 hours. At the end of incubation, supernatants were carefully collected from wells and transferred to sterile 1.5 ml Eppendorf tubes (Axygen) then frozen at −20°C.

### TSLP ELISA

Levels of human TSLP (hTSLP) in epithelial cell culture supernatants were measured with a Human TSLP ELISA development kit (Biolegand, UK) according to manufacturer's instructions.

### Generation of dendritic cells

Dendritic cells were generated from peripheral blood-monocytes as described before [30]. Briefly, human peripheral blood mononuclear cells (PBMC) (obtained after informed consent and following ethical committee approval) were separated by standard density gradient centrifugation on Histopaque (HISTOPAQUE-1077, Sigma, Irvine, UK). Purified PBMCs were incubated with mouse anti-human CD14^+^ monoclonal antibody conjugated to magnetic beads (Miltenyi Biotec, Surrey, UK). Cells were then washed and applied onto a column placed in the magnetic field of a MACS separator (Miltenyi Biotec, Surrey, UK). CD14+ cells (monocytes) were cultured (1×10^6^ cells per ml) in 48-well flat-bottomed culture plates (Costar, High Wycombe, UK) in RPMI-1640 medium supplemented with L-glutamine, antibiotics (Sigma, Irvine, UK) and 10% fetal calf serum (FCS, Harlan Sera-Lab, Loughborough, UK) containing 50 ng/ml of granulocyte-macrophage colony stimulating factor (GM-CSF) and 250 U/ml of IL-4 [DC-medium] (R&D Systems, Oxford, UK) at 37°C in 5% CO_2_ for 6 days.

### Allergen/non-allergen uptake and inhibition assays

DCs were washed and re-suspended in uptake medium consisting of 70% RPMI (RPMI 1640, Sigma, Irvine, UK), 25% PBS (Sigma, Irvine, UK) with Ca^2+^ and Mg^2+^ and 5% FCS (FCS, Harlan Sera-Lab, Loughborough, UK). Natural Der p 1 (nDer p 1) preparations were labelled with Cy5 (GE healthcare, Bedford, UK) in some experiments and FITC in others (Novus Bio, Cambridge, UK), recombinant Der p 1 (rDer p 1) and Staphopain B were labelled with FITC. In the uptake assays, cells were pre-incubated with natural or recombinant Der p 1 preparations (0.5 to 20 µg/ml) and their deglycosylated counterparts or Staphopain B (1.0 µg/ml), which is not an allergen. In the inhibition assays, mannan (200 µg/ml), galactose-PAA (200 µg/ml) and rDer p 1 (0.5 to 20 µg/ml) were incubated with the DCs for 20 mins at 37°C before the addition of Der p 1 followed by incubation for another 25 mins at 37°C. The uptake of labelled natural and recombinant Der p 1 was then immediately determined by flow cytometry using a Beckman–Coulter Altra flowcytometer (Beckman-Coulter, High Wycombe, UK) and expressed as mean fluorescence intensity (MFI). At least 10,000 cells per sample were analysed.

### Confocal imaging

Day 6 immature DCs were collected and washed with warm RPMI. Natural and recombinant (Indoor Biotechnology) Der p 1 preparations were labelled with FITC (Novus Bio, Cambridege, UK) and Cy5 (GE Healtcare, Bedford, UK), respectively. The cells were then incubated at RT for 5, 10, 15 or 30 mins with labelled allergens (1.0 µg/ml) prior to fixation with 4% formaldehyde and permeabilised with 0.1% triton X. The following antibodies and labelling reagents were used for cell staining: anti-MR (CD206) (PE; Clone 3.29B1.10, Coulter Immunotech), anti-LAMP-2(Lysosomal-associated membrane protein 2) PE (Clone GL2A7, Bioquote) and Fluoro-Trap Fluorescein Labelling Kit [FITC] were used according to manufacturer's protocol (Novusbio, UK). This reaction was incubated for 30 mins at RT. To label the nucleus, DAPI stain (Thermo Scientific) was used. For imaging, samples were set up on poly-l-lysine coated slides, covered with cover slips and imaged by LSM 510 meta Confocal Laser Scanning Microscopes (Carl Zeiss International) at 40× and 60×. Negative controls (cells labelled with the fluorochromes only (PE, Cy5, FITC)) were used to set up the lasers for imaging. Co-localization and image analysis were done using the LSM 510 image browser program.

### Anti-Der p 1 5H8 ELISA

Different unlabelled Der p 1 allergen preparations (natural and deglycosylated) were used at concentration of 2 µg/ml. Maxisorb ELISA Plates (Nunc, Roskilde, Denmark) were coated overnight by the allergen preparations, blocked with TBS buffer (TBS, 1% BSA), washed, then 5H8 anti-Der p 1 biotinylated antibody (clone 5H8 C12 D8) Indoors Biotechnology, Warminster, UK) was added and incubated at 2 µg/ml for 2 hours at room temperature. The binding was then detected by incubation with Extra-avidin alkaline phosphatase conjugate diluted 1∶1000 in TBS buffer (Sigma-Aldrich, Irvine, UK). Afterwards, plates were developed with 100 µl/well of (1 mg/ml) pNPP (Sigma-Aldrich, Irvine, UK). Absorbance was measured at 405 nm on a plate reader (Multiskan Ex, Labsystems, Helsinki, Finland). All assays were carried out in triplicate.

### MR binding

All washes and incubations were carried out in lectin buffer consisting of 10 mM Tris-HCl, pH 7.5, 10 mM Ca^2+^, 0.154 M NaCl and 0.05% (w/v) Tween 20. Different Der p 1 glycoforms at concentration of 2 µg/ml Der p 1 (Indoor Biotechnology), as well as 2 µg/ml of the corresponding carbohydrate ligand [Mannan (Sigma-Aldrich, Irvine, UK) or Galactose (Gal-PAA) (Lectinity, Moscow, Russia)] were used to coat the wells of Maxisorb ELISA plates (Nunc, Roskilde, Denmark) by overnight incubation in PBS at 4°C. The MR subfragment (CTLD4-7-Fc) (kindly provided by Dr Luisa Martinez-Pomares, University of Nottingham, UK) was then added at 2 µg/ml and incubated for 2 hours at room temperature. The binding was detected by incubation with anti-human IgG gamma-chain-specific alkaline phosphatase conjugate diluted 1∶1000 in the lectin buffer. Afterwards, plates were developed with 100 µl/well of (1 mg/ml) pNPP (Sigma-Aldrich, Irvine, UK) as a phosphatase chromogenic substrate. Absorbance was measured at 405 nm on a plate reader (Multiskan Ex, Labsystems, Helsinki, Finland). All assays were carried out in triplicate.

### Statistical analysis

Statistical analysis of the data was carried out using Student's t-test and *P-values*<0.05 were considered significant. Flow cytometry data were expressed as MFI ± SEM; number of independent experiments ≥3.

## Results

### Detecting the pattern of N- and O-glycosylation in allergens

Using GNA, SNA, PNA, MMA and DSA labelled lectins and anti-1,3 fucose antibody, allergens were assessed for the presence of different sugar moieties. Der p 1, a cysteine protease allergen from *Dermatophagoides pteronyssinus*, reacted with GNA which recognises 1–2,3 and 1–6 mannose, suggesting that it has high mannose N-glycans in its natural form [15]. Der p 1 also showed a positive reaction with anti-1,3 fucose (Table 1), which indicates that it has part of the CCD 1,3 fucose on its N-glycosylation site which is linked to asparagine. It also reacted with DSA, PNA and SNA, which respectively recognise 1,4 galactose, 1,3 galactose and sialic acid linked 2–6 to galactose. Der p 1 failed to react with MMA, thus suggesting that it does not contain any sialic acid binding to 2–3 galactose.

The recombinant preparation of Der p 1 that is produced in *Pichia pastoris* reacted with GNA to a higher degree than the natural preparation. The band itself was diffused, suggesting hyperglycosylation and its positive reaction with GNA confirmed that most of the glycosylation is due to mannosylation which is expected as proteins expressed in yeast tend to be hypermannosylated [24], [31]–[34]. The preparation also reacted with PNA suggesting that it also has some 1,3 galactose.

Unlike natural Der p 1, the recombinant preparation does not have any sialic acid or 1,4 galactose.

Fel d 1, the major cat allergen *Felis domesticus*, is shown here to have low levels of mannan as well as showing strong reactions with DSA and MAA, thus suggesting that it has 1,4 galctose and sialic acid (Table 1). It does not, however, contain 1,3 fucose which is expected as 1,3 fucose is not present in mammals. The recombinant deglycosylated counterpart of Fel d 1 does not contain any mannan, but it does contain sialic acid and 1,3 galactose (Table 1). Can f 1, the dog allergen *Canis familiaris*, is also not fucosylated as it is from a mammalian source [34], [35], but it is mannosylated and appears to contain sialic acid too (Table 1). The two allergens that showed a very strong reaction with mannan, in addition to rDer p 1, were Ara h 1 (*Arachis hypogaea*) and Bla g 2 (*Blattella germanica*). These two allergens are highly mannosylated in their natural forms (Table 1).

The other major house dust mite allergen, Der p 2, failed to give a positive reaction with GNA, indicating the lack of mannan in this allergen. It did, however, react with 1,3 fucose and also reacted positively with DSA, MMA, PNA and SNA, which suggest the presence of other carbohydrate moieties.

### The mannosylation patterns of allergens and non-allergens

**Figure 1:**
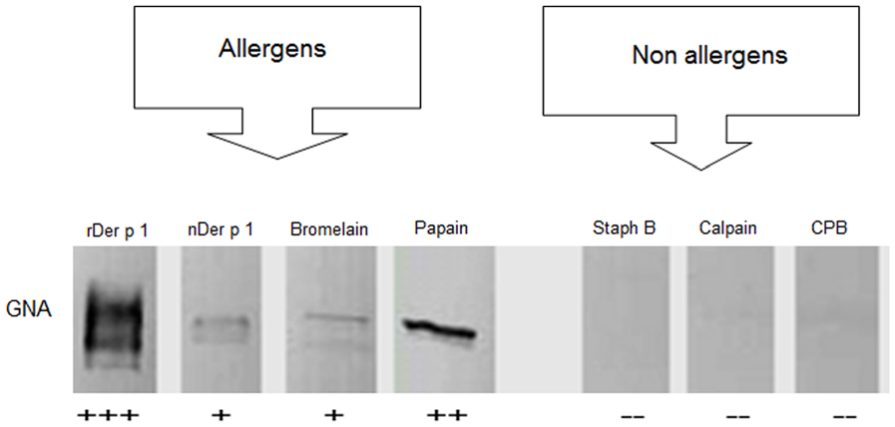
Comparative analysis of cysteine protease allergens and non-allergens in terms of mannosylation. Allergens are strongly mannosylated and have stronger reaction with anti-mannose GNA compared to non-allergens. +++: strong reaction, ++: moderate reaction, +: mild reaction, −: no reaction.

Our data indicate that mannosylation appears to be a dominant feature among allergens (Table 1). Therefore, a comparative carbohydrate analysis was done for proteins that are not known to elicit allergic responses (Staphopain B, Calpain and Cysteine Protease B (CPB) [36]–[38], yet share the same protein family with allergens such as Papain, Bromelain and Der p 1 [39]–[41]. All these proteins have potent cysteine protease activity, but very little is known about their glycosylation pattern. Following experiments using GNA lectin it became clear that the non-allergens Staphopain B, Calpain and CPB do not react with GNA, thus indicating that unlike allergens they are not mannosylated (Fig. 1). Staphopain B also did not react with any of the other lectins, indicating that it does not have any mannose, galactose or sialic acid.

### Comparative analysis of natural Der p 1, recombinant Der p 1 and Staphopain B uptake by immature DCs

**Figure 2:**
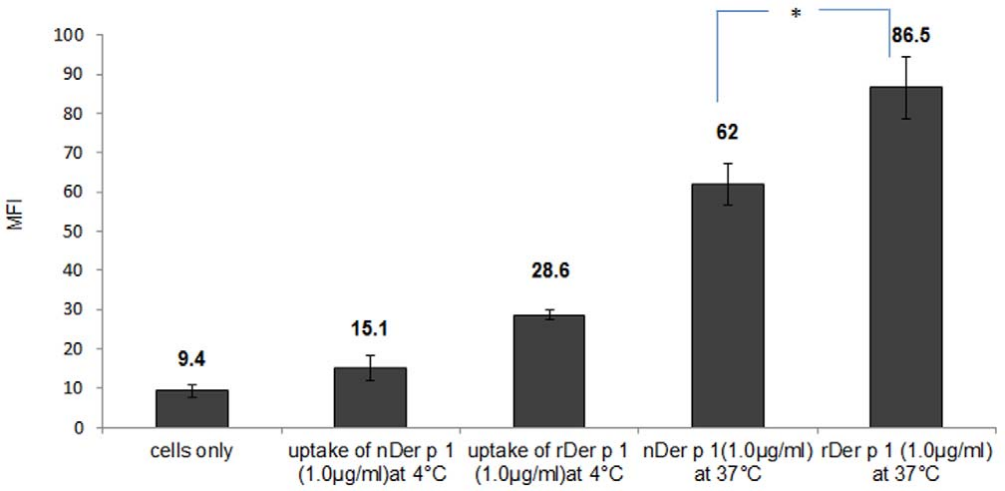
MFI ± SEM readings which represent the difference in uptake between natural and recombinant Der p 1 (1 µg/ml) by immature DCs. There was a significant difference between natural and recombinant allergen uptake. The results suggest that the average mean of uptake for the recombinant preparation is higher than that for natural Der p 1. The results also show that the uptake of Der p 1 by immature DCs at 4°C is lower than the uptake at 37°C for both preparations. Both natural and recombinant Der p 1 were labelled with FITC. *P value<0.05.

**Figure 3:**
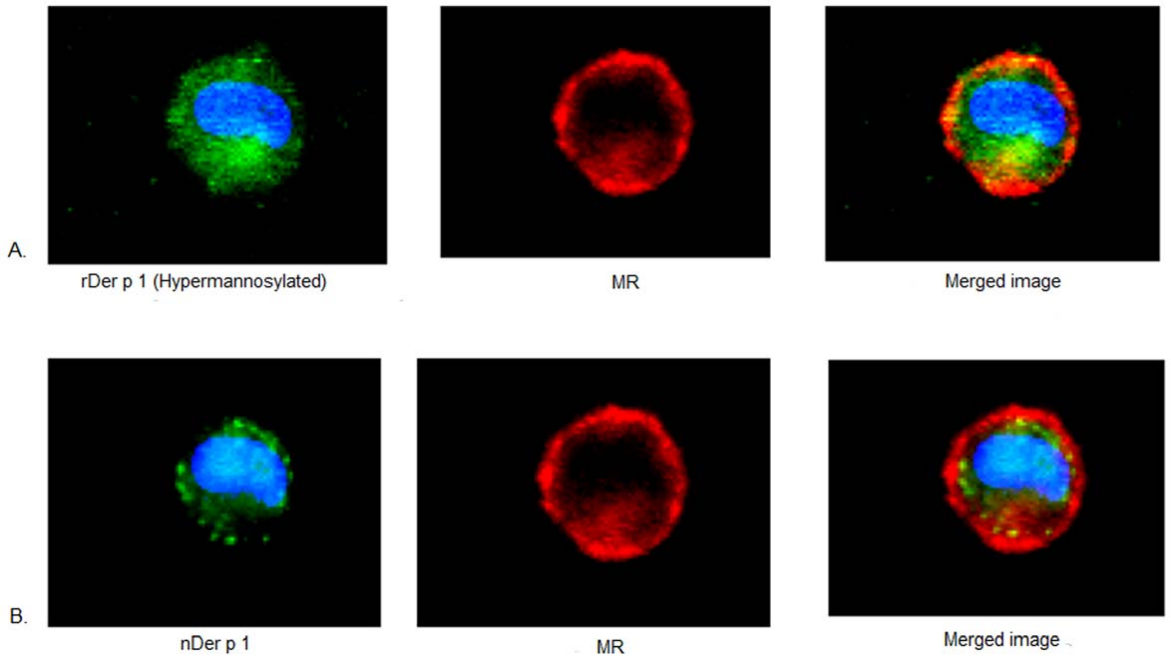
Confocal images of the difference between recombinant and natural Der p 1 (0.5 µg/ml) uptake by the same immature DC at 37°C. The results suggest that the uptake of the recombinant preparation (A) is higher than that for natural Der p 1 (B) in the same DC. A. Green: rDer p 1 labelled with FITC, red: MR labelled with PE, blue: nucleus labelled with DAPI. B. Green: nDer p 1 labelled with Cy5, red: MR labelled with PE, blue: nucleus labelled with DAPI.

**Figure 4:**
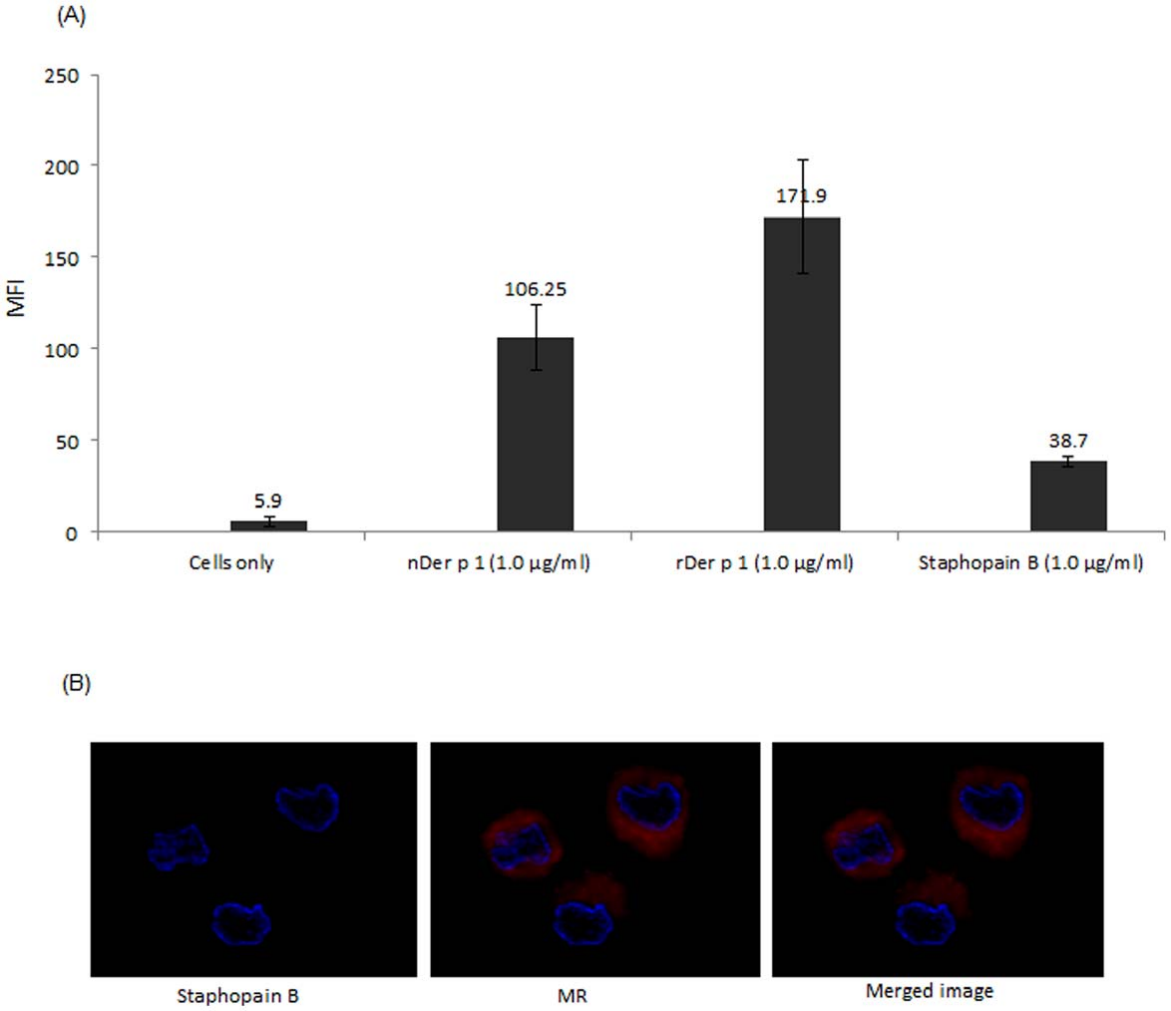
A. Natural and recombinant Der p 1 uptake by immature DCs at 37°C compared to the non-allergen Staphopain B at the same conditions and concentrations. Results presented as MFI ± SDM and all preparations were labelled with FITC. B. Confocal images of the uptake of Staphopain B by immature DCs.

Both natural and recombinant (hyperglycosylated) Der p 1 preparations are glycosylated albeit to different degrees. Staphopain B is also a cysteine protease protein, like Der p 1, but was shown to be an amannosylated antigen. To investigate the effect of glycosylation on the uptake of Der p 1 by DCs, we incubated all preparations under the same conditions with immature DCs at 37°C. All preparations were labelled by FITC and thus the uptake could be measured comparatively by flow cytometry as MFI readings. The control conditions for these experiments were DCs incubated with allergens at 4°C and DCs only. Levels of allergen uptake for each condition is presented as MFI (Fig. 2) and is also visualised using confocal imaging (Fig. 3). The results suggest that the average mean of uptake for recombinant Der p 1 (hyperglycosylated) is significantly (*P value<0.05) higher than that of natural Der p 1 at any given time point. We also studied the uptake of Staphopain B antigen, which is not known to induce any allergic reactions and is not mannosylated, and the results show minimal uptake of this non-allergen compared to Der p 1 (Fig. 4A & B).

**Figure 5:**
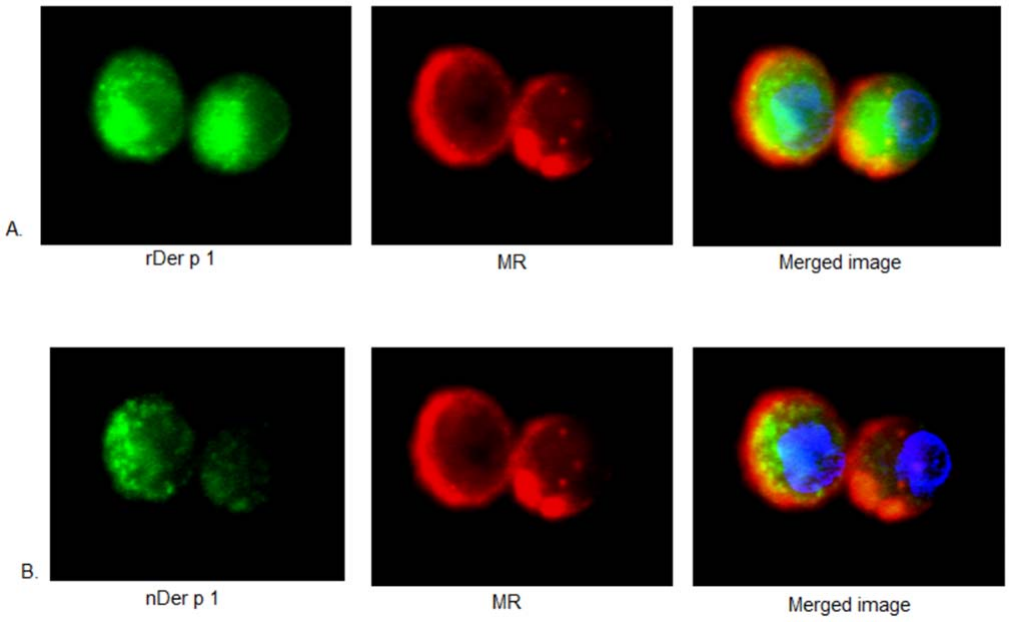
The uptake of recombinant and natural preparations of Der p 1 (1 µg/ml) by immature DCs at 30 mins. A. Green: rDer p1 stained with FITC, red: MR stained with PE, blue: nucleus stained with DAPI. B. Green: nDer p 1 stained with Cy5, red: MR stained with PE, blue: nucleus stained with DAPI.

**Figure 6:**
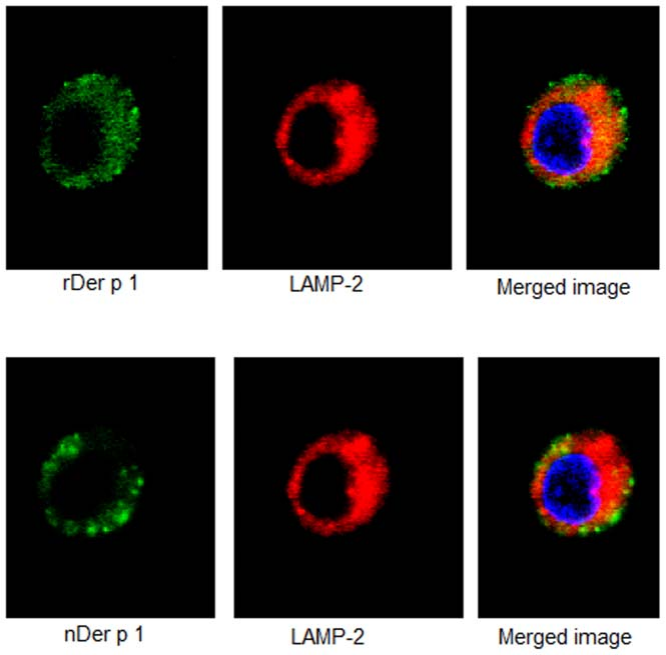
The co-localization of natural and recombinant Derp1 (0.5 µg/ml) with LAMP-2 detected at 10 mins. A. Green: rDer p 1 stained with FITC, red: LAMP-2 stained with PE, blue: nucleus stained with DAPI. B. Green: nDer p 1 stained with Cy5, red: LAMP-2 stained with PE, blue: nucleus stained with DAPI.

The confocal images also showed the co-localization of both Der p 1 preparations with MR (Fig. 5), although the co-localization coeffecient was found to be higher for rDer p 1 (0.911 compared to 0.84 for nDer p 1). Recombinant and natural Der p 1 also co-localised with the Lysosomal-associated membrane protein 2 (LAMP-2), which shuttles between lysosomes, endosomes and the plasma membrane (Fig. 6).

### Sodium periodate deglycosylation of natural and recombinant Der p 1

**Figure 7:**
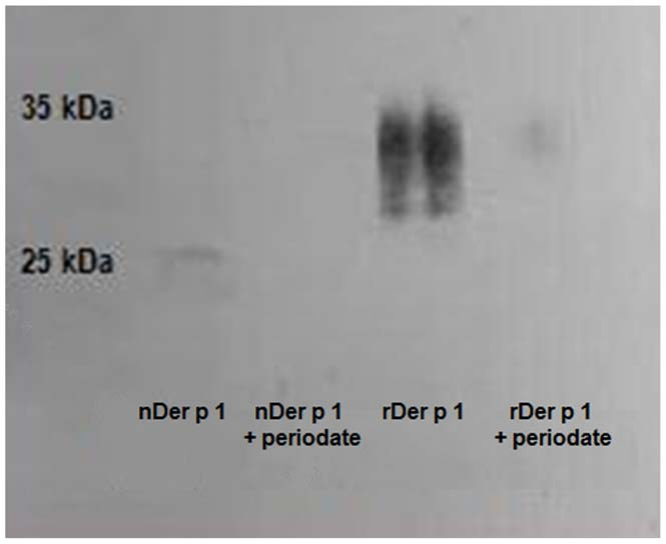
Western blot against GNA (anti-mannose) of natural and recombinant Der p 1 before and after periodate treatment. The blot shows minimal reaction with GNA for both preparations after periodate treatment, indicating that periodate removed most of the mannan. The concentration of the protein loaded in each well was 2.0 µg/ml.

**Figure 8:**
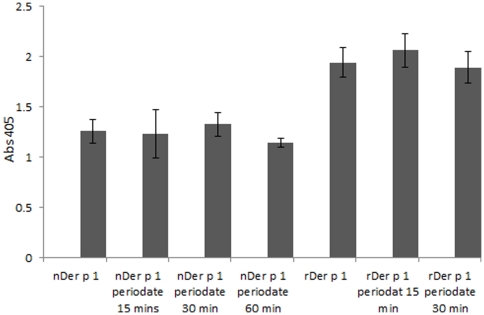
ELISA experiments showing the binding of natural Der p 1 and recombinant Der p 1 to anti-Der p 1 5H8 antibody before and after deglycosylation with periodate. Der p 1 was used at the same concentration (2.0 µg/ml) for all conditions. Data show the average of triplicate experiments.

**Figure 9:**
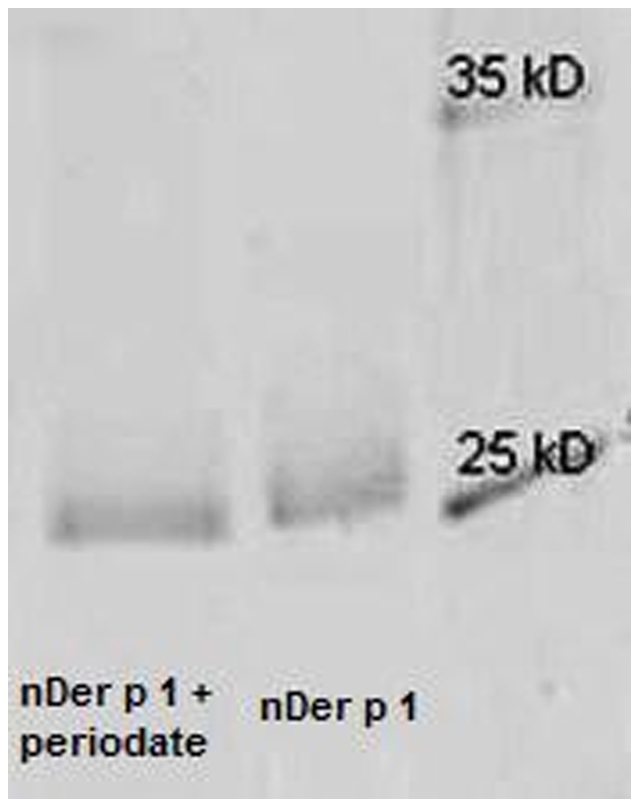
Commassie blue stained gel of natural Der p 1 before and after deglycosylation with periodate showing a slight decrease in the MW of deglycosylated Der p 1.

**Figure 10:**
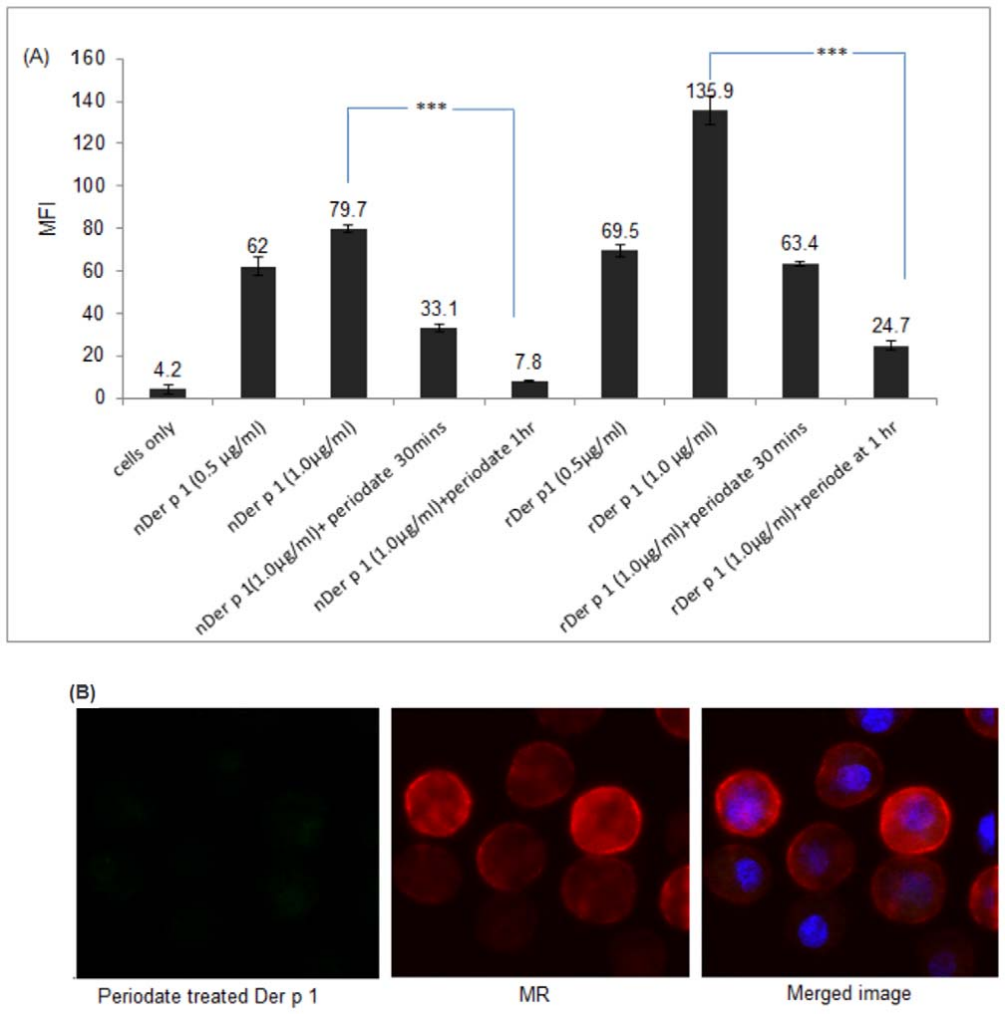
A. The MFI ± SEM readings for the uptake of different concentrations of nDer p 1 and rDer p 1 by immature DCs compared to the periodate treated preparations. Both nDer p 1 and rDer p 1 were treated with periodate for 30 mins and 1 hr, then their uptake was measured. The results show a significant decrease in uptake of periodate treated preparations compared with the untreated ones.*** *P value*<0.001, all Der p 1 preparations were labelled with FITC. B. Confocal images showing the uptake of periodate treated Der p 1 by immature DCs.

Periodate oxidation was used to deglycosylate both natural and recombinant Der p 1 preparations by using sodium metaperiodate. Periodate has been used in the literature to deglycosylate protein preparations [42]–[45] and it is known to remove mannose and fucose from proteins. Natural and recombinant Der p 1 were incubated with periodate in the dark at room temperature for 30 and 60 mins. A western blot against GNA (anti 1–2,3,6 mannose) was performed on the samples before and after periodate treatment (Fig. 7) to confirm that demannosylation had worked. All these glycoforms retained their reactivity with anti-Der p 1 5H8 monoclonal antibody (Fig. 8), thereby ascertaining their structural integrity. A commassie blue stained gel of natural Der p 1 before and after deglycosylation with periodate showed a slight decrease in deglycosylated Der p 1 MW as to be expected (Fig. 9). The preparations were then labelled with FITC and the uptake by DCs was measured against untreated preparations (Fig. 10 A).

The results indicate a significant decrease in the uptake of both Der p 1 preparations after periodate treatment. The periodate treated recombinant preparation (1 µg/ml) showed a 53% decrease in uptake after 30 mins of treatment compared to the untreated preparation of the same concentration; at 60 mins of periodate oxidation the uptake decreased to 81.6%. The periodate treated natural sample showed a decrease of 58.7% after 30 mins of treatment and 90% decrease in uptake after 60 mins (Fig. 10 A). We also used confocal microscopy to detect the uptake of periodate treated natural Der p 1. This showed almost complete abrogation of Der p 1 uptake after 60 min periodate oxidation (Fig. 10 B).

### MR binding by the different glycoforms of Der p 1

**Figure 11:**
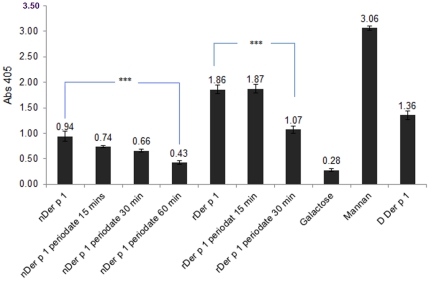
Binding of MR C-type lectin-like carbohydrate recognition domains 4–7 with different glycoforms of Der p 1, nDer p 1 and rDer p 1 (concentrations at 2 µg/ml). ***P value<0.001.

In order to study the effect of glycosylation on Der p 1 recognition by MR, binding to MR subfragment CTLD 4–7, the C-type lectin carbohydrate recognition domain which has been shown to be the main Der p 1 binding site, was investigated using ELISA. Mannan and galactose were used as positive and negative controls, respectively. Results in Fig. 11 show a significant decrease in binding to MR (>55%) when Der p 1 was deglycosylated with periodate for 60 mins. The same effect was also seen with hypermannosylated rDer p 1, as the decrease in binding after deglycosylation reached 42.5%. It is clear that the binding of the recombinant preparation of Der p 1 to MR is much stronger than that of natural Der p 1, which is expected since rDer p 1 has more mannan than its natural counterpart.

### Differences in TSLP secretion induced by the different glycoforms of Der p 1

**Figure 12:**
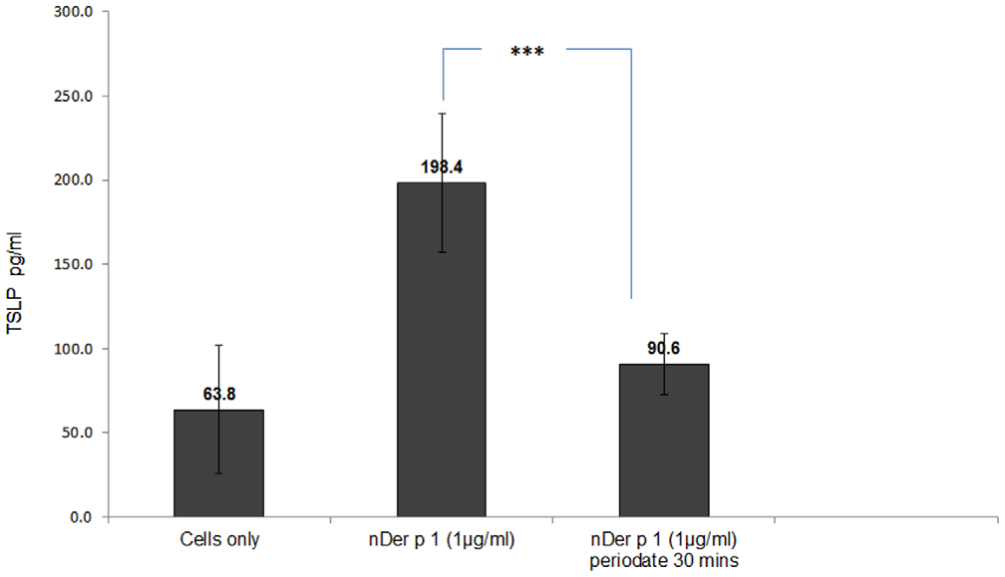
Differences in TSLP secretion in human BEAS-2B epithelial cells after 24 hrs stimulation with different glycoforms of Der p 1. Concentration of all Der p 1 preparations used was 1 µg/ml. ***P value<0.001.

Different glycoforms of Der p 1 were incubated with a human epithelial cell line (BEAS-2B) for 24 hrs followed by TSLP measurement in the supernatants. Results in Fig. 12 show a significant increase in TSLP secretion by human epithelial cells when challenged by Der p 1, with lower TSLP production in response to deglycosylated Der p 1.

## Discussion

Glycosylation in allergens is a key structural feature and mannosylation seems to be the dominant glycosylation pattern with the exception of Der p 2, which possesses galactose, sialic acid and N-acetylglucosamine. We have shown the predominance of mannan in some of the most dominant environmental allergens such as Ara h 1, Bla g 1, Can f 1, Fel d 1, Bromelain and Papain. We also showed that mannosylation is absent in non-allergen proteins that are structurally similar to cysteine protease allergens. Other groups reported the presence of mannan in Cedar allergen Cry j 1 [46], pollen allergen Cha o 1 [47], yellow jacket allergen Ves v 2 [48] and Ovalbumin [49]. Ara h 1, Cor a 11, Jug r 2 and Ana o 1 allergens have been reported to contain a xylose and mannose in the *N*-glycan chain [50], [51]. Fucose 1,3 is reported to be present in a wide range of allergens like Hev b 1, Ara h 1, Bromelain and Papain [29], [35], [52]. The degree of mannosylation clearly differs between allergens like Der p 1, Papain, Bromelain and their structurally similar non-allergen counterparts like CPB, Calpain and Staphopain B.

The detection of galactose 1,3, galactose 1,4, sialic acid and 1,3 fucose in allergens provided for the first time a better insight into the structure of carbohydrates in allergens. Although most reports concentrate on N-glycosylation as a target for lectin receptors on antigen presenting cells, some recent reports did suggest that O-glycosylation by itself plays a role in CCD [53]–[55], which is why it is important to be comprehensive in studying glycans on allergens and determining the specific structures of both O- and N-glycans in them.

The recombinant hypermannosylated Der p 1 preparation used in this study was taken up more readily by DCs than natural Der p 1, and this underlines the importance of sugars in allergen recognition by C-type lectin receptors like MR and DC-SIGN [15], [19], [56], [57]. We have previously shown that Der p 1 binding to MR, most likely through regulation of indoleamine 2,3 dioxygenase (IDO) activity, plays a key role in down stream allergen induced Th2 cell differentiation [15]. Given the commonality of mannosylation amongst allergens from diverse sources, it is reasonable to suggest that allergen glycosylation plays a central role in their allergenicity. Within this context, it is therefore not surprising that the non-allergen Staphopain B, which is not mannosylated, is not taken up efficiently by immature DCs compared to Der p 1.

The above data were further corroborated by demannosylating Der p 1 via chemical deglycosylation resulting in a preparation exhibiting minimal uptake by DCs. This was best exemplified by sodium metaperiodate treatment of Der p 1. Sodium metaperiodate treatment does not affect the protein conformation under mild conditions, and it was shown that at 10 mM will lead to alterations in carbohydrates structure without any significant effect on proteins integrity in *Schistosoma mansoni* Egg antigens [45]. Periodate was also used to destroy carbohydrates on Cry j 1, the major allergen of Japanese cedar pollen, and it was shown that after periodate oxidation Cry j 1-specific CD4+ T cell proliferation decreased significantly and there was also significantly less IL-4 and IL-5 secretion in comparison with the control antigen [58]. Consequently, those authors suggested a role for carbohydrates in Cry j 1 in promoting Th2 immune responses *in vitro*.

The confocal images provided an insight into the uptake of different Der p 1 preparations by DCs. It became clear that the internalization of Der p 1 is initiated by MR on immature DCs. Despite, different rate of uptake by DCs, both recombinant and natural Der p 1 co-localised with LAMP-2, a lysosomal marker, suggesting a common fate for these preparations inside the DC.

Epithelial cells provide the initial barrier for defence against allergens. The epithelial barrier in the skin, gastrointestinal tract and airways plays an important role in initiating immune responses by secreting chemokines, cytokines and growth factors like IL-1, IL-6, IL- 8, GM-CSF, Interferon α and β, TNF-α and others which provoke immune and inflammatory reactions [5], [10], [59]. Epithelial cells also produce TSLP in response to allergen exposure. This cytokine was originally described in B cell proliferation and development [60]. Since then, TSLP has been described to target and regulate numerous DC and monocyte activities. Several studies concluded that TSLP drives DC maturation for Th2 immune responses via enhancing pro-allergic Th2 type cytokines like IL-4, IL-5 and IL-13 [6], [10], [61] and up-regulating the co-stimulatory molecules CD40, CD80, CD83 and CD86 [10], [60]–[65].

House dust mite allergens have been shown to induce TSLP production by epithelial cells [66], [67]. This was confirmed by our data showing a significant increase in the secretion of TSLP by epithelial cells in response to Der p 1 compared to the non-allergen controls. Interestingly, the level of TSLP production in response to Der p 1 stimulation seems to be positively related to the level of allergen glycosylation, since when challenging human epithelial cells with a deglycosylated preparation of Der p 1, the TSLP secretion was significantly reduced. This may therefore indicate the presence of lectin like receptors on epithelial cells and a role for carbohydrates in recognition of allergens by the epithelia.

In conclusion, this work progresses the definition of allergenicity and correlates it to the glycosylation pattern of allergens. Thus, it appears that glycosylation is a key feature of many allergens and that mannan seems to be the dominant sugar moiety associated with allergens [15], [68], [69]. Therefore, it is now tempting to suggest that the counter structures of these carbohydrates on innate immune cells, namely MR and other C-type lectin receptors, could potentially be targeted to stop allergen uptake at the point of initial contact with innate body defences. Alternatively, developing different glycoforms of allergens with ‘Immune-regulatory’ properties could prove to be a useful strategy in allergen-specific immunotherapy approaches [70], [71].
